# Figural Memory Impairment in Conjunction With Neuropsychiatric Symptoms in IgLON5 Antibody-Associated Autoimmune Encephalitis

**DOI:** 10.3389/fpsyt.2020.00576

**Published:** 2020-07-03

**Authors:** Niels Hansen, Sina Hirschel, Winfried Stöcker, Anja Manig, Hannah Sönne Falk, Marielle Ernst, Ruth Vukovich, Inga Zerr, Jens Wiltfang, Claudia Bartels

**Affiliations:** ^1^ Department of Psychiatry and Psychotherapy, University Medical Center Goettingen, Goettingen, Germany; ^2^ Euroimmun Laboratory, Seekamp, Luebeck, Germany; ^3^ Department of Clinical Neurophysiology, University Medical Center Goettingen, Goettingen, Germany; ^4^ Department of Neuroradiology, University Medical Center Goettingen, Goettingen, Germany; ^5^ Neurochemical Laboratory, Department of Neurology, University Medical Center Goettingen, Goettingen, Germany; ^6^ German Center for Neurodegenerative Diseases, Goettingen, Germany; ^7^ Neurosciences and Signalling Group, Department of Medical Sciences, Institute of Biomedicine, University of Aveiro, Aveiro, Portugal

**Keywords:** encephalitis, IgLON5 antibodies, memory impairment, figural memory, autoimmunity

## Abstract

**Background:**

IgLON5 disease is an autoimmune disorder that shares neuropathological aspects with a tauopathy. Its clinical spectrum is heterogeneous, and figural memory impairment as an initial phenomenon of IgLON5 syndrome has not yet been described. The rationale of this report is to highlight symptoms related to IgLON5 disease that have not been reported to date. This case report will thereby emphasize how important it is to initiate thorough diagnostic methods including cerebrospinal fluid analysis (CSF) before starting early immunotherapy.

**Methods:**

We examined a 65-year-old Caucasian male *via* neuropsychological tests, magnetic resonance imaging (MRI), electroencephalography (EEG), neurography and polysomnography. He also underwent two lumbar punctures from which we determined specific autoantibodies in cerebrospinal (CSF) and peripheral blood (PB).

**Results:**

The patient presented initially complaining of memory loss, gradual dysphagia and sleeping dysfunction. Neuropsychological testing at first presentation and follow-up revealed subtle figural and working memory impairment. At onset and at his 6-month follow-up, we detected IgLON5 antibodies in CSF and PB. Furthermore, we identified in the CSF a blood–brain barrier disturbance at disease onset and follow-up, and markers of neuroaxonal damage such as mildly elevated phosphorylated Tau-181 protein with 86 pg/ml (normal range ≤ 61 pg/ml) at onset. Three months after his initial presentation, he was suffering from axonal neuropathy and transient ataxia in the extremities. Assuming a definitive autoimmune encephalitis-associated with anti-IgLON5 antibodies, we applied high-dose steroids monthly (1g methylprednisolone i.v. for five consecutive days) and his memory complaints, ataxia of extremities and peripheral neuropathy as well as sleeping dysfunction decreased.

**Conclusions:**

Our findings broaden IgLON5 disease’s clinical spectrum to include predominant and discrete figural memory impairment together with sleeping dysfunction at disease onset. In addition, our report illustrates how important taking an elaborated diagnostic approach is to assuring an accurate diagnosis and the appropriate therapy if a patient presents with a persisting figural memory impairment and sleeping abnormalities so as to avoid overlooking IgLON5 disease and a potentially poor outcome.

## Introduction

IgLON5 antibody-associated encephalitis is a rare autoimmune-mediated disorder of the central nervous system often presenting with a tauopathy. Various clinical features have been described about anti-IgLON5 antibody-mediated encephalitis, such as sleep dysfunction (e.g. sleep-apnea, insomnia, and/or parasomnia), brainstem and/or cerebellar dysfunction (e.g. ataxia, abnormal ocular movements, dysphagia, or respiratory dysfunction), and neuropsychiatric symptoms like cognitive dysfunction (1, 2). The IgLON5 antigen is a cell-adhesion molecule whose functions are incompletely understood. We here describe for the first time the clinical case of a male patient presenting a subtle figural memory impairment in conjunction with numerous subjective cognitive complaints and additional neuropsychiatric symptoms associated with cerebrospinal fluid (CSF) anti-IgLON5 autoantibodies as an early manifestation of IgLON5 disease. This report’s rationale is to highlight the subtle figural memory impairment together with other neuropsychiatric features as a relevant indicator for taking a sophisticated diagnostic approach encompassing the investigation of IgLON5 autoantibodies in CSF. We believe that the decision for initiating elaborated diagnostic methods in our case was essential, as the mortality of IgLON5 encephalitis can be high (1, 3).

## Patient and Methods

We examined a 65-year-old Caucasian male patient who presented in our tertiary memory clinic. The patient is married and father of two children. He has an educational level of 13 years and works as an administration employee. Our diagnostic schedule comprised comprehensive neuropsychological assessment, electroencephalography (EEG), magnetic resonance imaging (MRI), peripheral blood (PB) and cerebrospinal fluid (CSF) analyses including specific antibodies and markers of neuronal degeneration, polysomnography (PSG) and neurography. Neuropsychological testing and follow-up after six months – split into two sessions – covered all major cognitive domains (language, attention, executive functions, visuoconstruction, and memory) and comprised the following instruments: Beck Depression Inventory (BDI-II), CDT (Clock Drawing Test), CERAD (consortium to establish a registry for Alzheimer´s disease)-plus, Mini-Mental Status Examination (MMSE), Regensburger word fluency test (RWT), Rey-Osterrieth-Complex-Figure test (ROCFT), and subtests from the Wechsler Adult Intelligence Scale IV (WAIS-IV) and Wechsler Memory-Scale IV (WMS-IV). We sought specific antibodies against paraneoplastic antigens *via* antibody blots [Amphiphysin, CV2 (cronveinten 2), GAD65 (glutamic acid decarboxylase 65), HuD, Ma1/2, Ri, Ro, SOX1, TR, Zic4] in CSF and PB. Furthermore, we did antibody testing of CSF and PB with recombinant-cell indirect immunofluorescence against neuronal antigens such as Aquaporin-, AMPAR1/2- (α-amino-3-hydroxy-5-methyl-4-isoxazolepropionic acid receptor 1/2), CASPR2- (contactin-associated protein-like 2), DPPX- (dipeptidyl peptidase protein-like 6), GABAAR- (γ-aminobutyric acid A receptor), LGI1- (Leucine-rich glioma inactivated 1) and NMDAR- (N-methyl-D-aspartate receptor) antibodies. These antibodies were screened in the CSF laboratory in the Department of Neurology, University Medical Center Goettingen, IgLON5 antibodies were analyzed in the Euroimmun laboratory in Luebeck, Germany. We obtained informed consent from our patient and ethical approval. Our report concurs with the Declaration of Helsinki.

## Results

**Figure 1:**
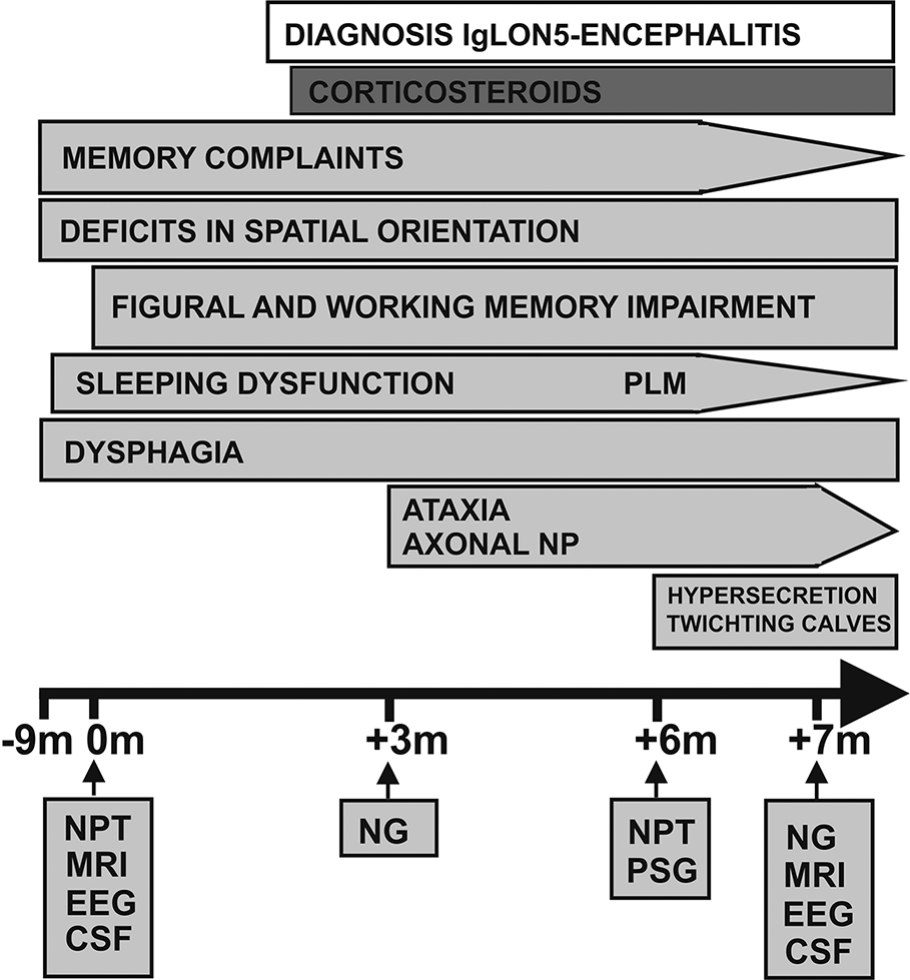
Time course of subjective and objective symptom evolution together with treatment. This schematic figure depicts the time course of symptoms from its first appearance, first presentation in hospital (onset) to their follow ups along with the performed diagnostic methods and corticosteroid treatment. CSF, cerebrospinal fluid analysis; EEG, electroencephalography; M, month; MRI, magnetic resonance imaging; NG, neurography; NPT, neuropsychological testing; PML, periodic limb movements; PSG, polysomnography; NP, neurography.

Our patient presented at first with a striking subjective memory decline. He reported having experienced difficulty finding words, naming things and persons. Both his forgetfulness and high distractibility often resulted in misplacing everyday items such as keys, as his wife reported. He had great difficulty concentrating, and experienced sleep disturbances (sleeping less than 3–4 h and waking up early). Furthermore, he reported mild dysphagia, and problems with spatial orientation in a novel environment during the previous 9 months (**Figure 1**). His wife also reported an increase in snoring over the last 9 months without its having interfered with his sleep. His daily living activities were unaffected. His medical history revealed no relevant comorbidities nor any relevant past or recurrent treatments. His mother suffers from parkinsonism. His physical examination exposed no pathological findings, but his neurologic examination revealed a known left-sided aniscoria. The patient found his cognitive deficits most afflicting; they had developed with a subacute onset, but no further deterioration. Cognitive screening results applying the MMSE and CDT were normal (MMSE=30/30, CDT=1), and the patient’s self-report indicated a euthymic affective state (BDI-II=4) (**Table 1**).

**Table 1:** Results of individual investigations at first visit and follow-up.

Parameter	First visit	Follow-up
**CSF analysis**		
Cell count /ul (pathological: >5 µl)	8	8
Lymphocytes in %	93	92
Monocytes in %	5	.
Plasma cells in %	2	2
Albumin mg/L	518	615
IgG mg/L	69.3	62.5
IgA mg/L	10.7	9.6
IgM mg/L	0.97	0.84
QAlb %	11.5	15.4
QIgG %	7.3	8.9
QIgA %	4.3	5.2
QIgM %	2.4	2.4
Lactat mmol/L	2	2
Oligoclonal IgG	–	–
NSE ng/ml (pathological: >30 ng/ml)	19.3	.
S100 µg/ml (pathological: >2.7 µg/ml)	2	.
Tau pg/ml (pathological: >450 pg/ml)	409	.
P-Tau 181 pg/ml (pathological: > 61 pg/ml)	68	.
Aß1-42 pg/ml (pathological: >450 pg/ml)	715	.
Aß1-40 pg/ml	9565	.
Aß ratio pg/ml (pathological: >0.5 pg/ml)	0.75	.
**Serum**		
Oligoclonal IgG	–	–
CRP mg/l	42.5	1.1
Leukocytes 10^3^ µl	5.63	4.29
Neuropsychological testing*		
MMSE	30	29
CDT	01	01
CERAD Boston Naming Test	PR 31	PR 82
CERAD semantic fluency	PR 50	PR 66
CERAD phonemic fluency	PR 82	PR 62
TMT part A	PR 76	PR 99
WAIS-IV Digit Symbol Test	PR 84	PR 95
TMT division part B/A	PR 84	PR 58
RWT semantic fluency (alternating)	PR 30	PR 75
RWT phonemic fluency (alternating)	PR 63	PR 54
WAIS-IV Digit Span forward	PR 05*	PR 05*
WAIS-IV Digit Span backward	PR 16	PR 16
CERAD List Learning (trials 1-3)	PR 34	PR 34
CERAD List Recall (savings)	PR 46	PR 54
CERAD List Recognition/discriminability	PR 21	PR 79
WMS-IV Logical Memory I	PR 50	PR 50
WMS-IV Logical Memory II	PR 16	PR 50
CERAD Figure recall (savings)	PR 01*	PR 12*
WMS-IV Visual Reproduction I	PR 25	PR 50
WMS-IV Visual Reproduction II	PR 50	PR 75
CERAD Figure Copy	PR 73	PR 18
ROCFT Copy	PR>16	PR>16
WAIS-IV Block Design	PR 25	PR 75
**Psychopathology**		
BDI-II	04	05
**Polysomnography**		
Apnoe-Hypopnoe-Index	.	9.7
PLMI	.	64.6
Basal 0_2_ desaturation %	.	94
Minimal 0_2_ desaturation %	.	89.0
0_2_ desaturation index %	.	13

*For better comparison, cognitive data is presented as percentiles (PR) unless otherwise indicated. Asterisk denote performance below the normal range (PR<16). . = data not available. - = not present. Polysomnography data are derived from the second night.

AHI, apnoe-hyperpnoe index; Aß ratio, ß amyloid 1-42/1-40 ratio; Aß1-40, ß amyloid 1-40; Aß1-42, ß amyloid 1-42; BDI-II, Beck Depression Inventory; CSF, cerebrospinal fluid; IgA, immunoglobulin A; IgG, immunoglobulin G; IgM, immunoglobulin M; MMSE, Mini-Mental Status Examination; CDT, Clock Drawing Test; CERAD, Consortium to Establish a Registry for Alzheimer’s Disease; TMT, Trail Making Test; RWT, ROCFT, Rey-Osterrieth-Complex-Figure test; WAIS-IV, Wechsler Adult Intelligence Scale IV; WMS-IV, Wechsler Memory-Scale IV, NSE, neuron specific enolase; O_2_ desaturation, oxygen desaturation; PLMI, periodic limb movement index; P-tau181, phosphorylated tau protein 181; QAlb, quotient albumin; QIgA, quotient immunoglobulin A; QIgG, quotient immunoglobulin G; QIgM, quotient immunoglobulin M; T-tau, total tau protein.

Most interestingly, cognitive impairment was detected in figural memory parameters in the CERAD-plus test battery, but not in the immediate and delayed recall in WMS-IV Visual Reproduction subtests, although the latter relies on more complex stimuli and entails a longer delayed recall interval. A slight impairment was also observed in his verbal memory span (WAIS-IV Digit Span forward), while all other cognitive functions appeared unaffected (**Table 1**). At neuropsychological follow-up six months later, we detected a similar pattern with stable, subtle impairments in figural memory and working memory (**Figure 1**, **Table 1**). Modest improvements in other cognitive parameters must be interpreted cautiously and while considering practice effects.

**Figure 2:**
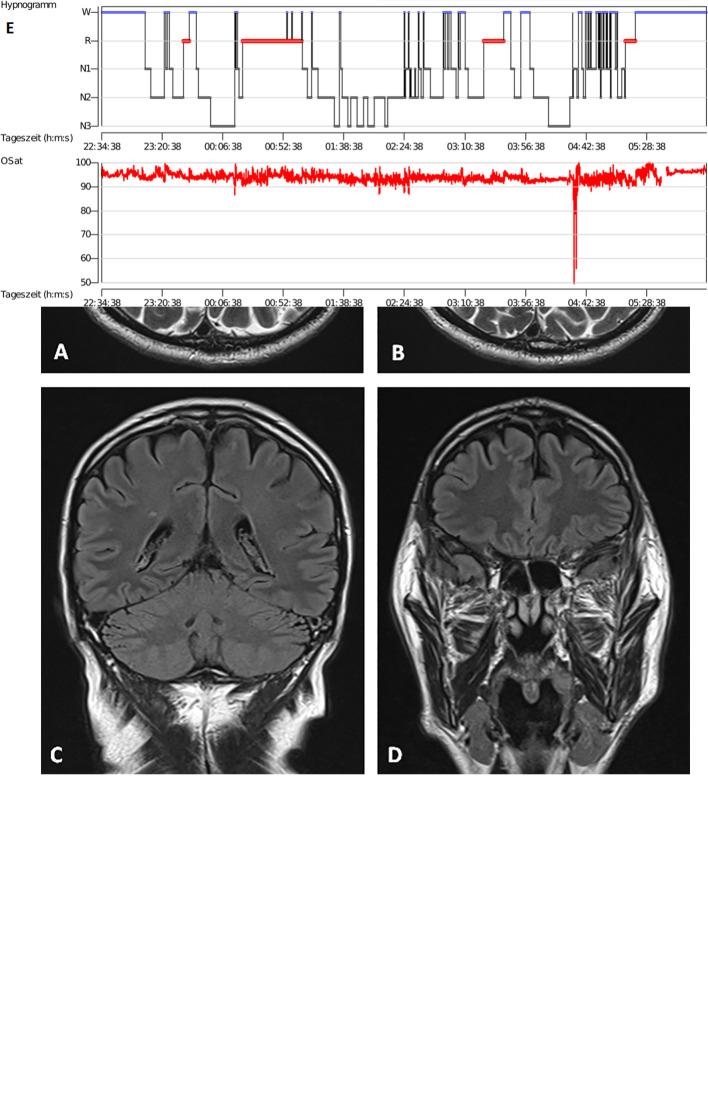
Brain magnetic resonance imaging and polysomnography. Brain magnetic resonance imaging (Siemens Avanto 1.5 T). Axial T2w **(A, B)** and coronal FLAIR **(C, D)**. Hyperintense periventricular and juxtacortical white matter lesions. No specific MRI findings for IgLON5-associated encephalitis. **(E)** In the upper graph the hypnogram is shown as a function of time of the day (hours: minutes: seconds) with W representing wakefulness, R representing rapid eye movement (REM) sleep and N1-3 representing non-REM-sleep stages 1 to 3. In the lower graph oxygen saturation measured *via* pulse oximetry is depicted as a function of time of the day (hours: minutes: seconds) ranging from 50 to 100%.

For differential diagnosis, we performed lumbar puncture, analyzed CSF, and detected a pleocytosis of leukocytes (for specifications see **Table 1**). Specific antibodies (see methods) in PB and CSF were not detected. Furthermore, we identified in the CSF a blood-brain barrier disturbance and markers of neuroaxonal damage such as mildly elevated phosphorylated Tau-181 protein with 86 pg/ml (normal range ≤ 61 pg/ml). Peripheral inflammation was indicated by elevated C-reactive protein (CRP) with 42.5 mg/l (normal range < 5 mg/l) and an elevated blood sedimentation rate (1h: 86/2h: 88 mm according to Westergren). MRI revealed frontal subcortical lesions on the right-side and a few unspecific periventricular white-matter lesions. EEG recordings showed neither epileptic potentials nor focal slowing (**Figure 2**).

CSF diagnostics revealed a pleocytosis, a blood–brain barrier disturbance together with mild unspecific neurodegeneration, elevated phosphorylated tau-protein, and negative standard panel of specific autoantibodies (see *Methods*, **Table 1**). Our patient had reported sleeping dysfunction in addition to his figural and working memory impairment. Sleeping dysfunction is a prominent symptom of IgLON5 disease (4–6). His clinical syndrome, which comprised cognitive deficits together with sleeping abnormalities in conjunction with signs of CNS inflammation was our rationale to search for anti-IgLON5 antibodies (Euroimmun Laboratory, Luebeck, Germany) beyond the standard antibody panel. Anti-IgLON5 antibodies in serum (1:320) and CSF (1:3200) were detected *via* indirect immunofluorescence assay. Finally, we diagnosed an autoimmune encephalitis associated with anti-IgLON5 antibodies according to the Graus criteria (7) and started high-dosage therapy with methylprednisolone (1g/d i.v. over five days), to be continued over 6 months with 5 days of corticosteroids administered monthly as first therapy of choice in autoimmune encephalitis. Two weeks after having starting this well-tolerated corticosteroid treatment with no report of any adverse event, the patient complained at follow-up of dysphagia that he had noticed for several months but which he had ignored until then. In addition, he reported at follow-up (and after 3 cycles of corticosteroids) that he had been sleeping better (for 6 h uninterrupted), although he still suffers daytime sleepiness. His subjective memory impairments were less pronounced at follow-up, but he complained further of increased hypersalivation, his calves twitching, and of worse subjective dysphagia at follow-up. An endoscopic examination of his throat revealed no cause for his dysphagia. We decided to continue monthly intravenous administration of high-dose corticosteroids due to his improved cognitive and sleeping function. A follow-up neuropsychiatric examination 3 months after his initial presentation revealed ataxia of extremities (**Figure 1**) that had disappeared by his final follow-up 7 months after initial presentation. In the meantime, he was diagnosed with axonal sensorimotor polyneuropathy *via* neurophysiologic measurements 3 months after his initial presentation (**Figure 1**), but another investigator could not confirm that finding neurographically at his last follow-up. PSG given over three nights 6 months after his initial presentation (**Figure 2**) revealed central (and mainly in supine position) obstructive sleep apnea and oxygen desaturation as low as 89% (**Table 1**, **Figure 2E**). However, after being given an additional pillow to discourage back sleeping, his obstructive apnea lessened. We also observed increased period limb movements (PLM) in PSG. At his last follow-up 7 month after initial presentation, MRI showed known frontal subcortical lesions on the right side and periventricular white matter lesions (**Figures 2A–D**) and a previously undescribed mild global atrophy. His last follow-up EEG still revealed no pathological signs. Subsequent CSF analysis showed a pleocytosis (**Table 1**) and a blood-brain barrier disturbance. Again, indirect immunofluorescence assay revealed anti-IgLON5 antibodies in serum and CSF at follow up.

## Discussion

With our patient’s cognitive testing results, the clinical IgLON5 syndrome’s spectrum is expanded to include figural memory impairment. Very few patients with IgLON5 antibody-positive encephalitis and cognitive dysfunction have been reported in the literature so far (1, 2, 8–11). This could be due to both the lack of neuropsychological testing and IgLON5 disease’s broad clinical spectrum—often presenting with predominant sleep dysfunction and motor impairment (9). So far, the cognitive spectrum of IgLON5 disease covers deficits in attention, information-processing speed, disinhibition and executive dysfunction, as well as impaired verbal memory performance (1, 2, 8–11), often progressing from mild cognitive impairment to dementia (10, 11). The pathophysiological mechanism of figural memory dysfunction in IgLON5 disease remains unclear. However, we hypothesize that IgLON5 autoantibodies result in phosphorylated Tau-deposits within the right hippocampus, as this structure is involved in figural memory function (12). A recent study demonstrated phosphorylated Tau-protein pathology in temporal regions including specific hippocampal subfields such as the cornu ammonis 1 (CA1) and the subiculum (13) concurring with a hypothetical mechanism for our patient’s figural memory impairment.

To monitor his figural memory impairment, we will therefore continue to examine this patient’s cognitive status. What is more, our patient’s concomitant features like insomnia, dysphagia, concomitant tau pathology, and mild CSF pleocytosis correspond closely with IgLON5 disease (1, 2). Our diagnostic approach entailing repeated CSF analysis providing stable proof of IgLON5 antibodies in the CSF delivers solid evidence for probable autoimmunity. In addition, the repeated confirmation of CSF antibodies together with signs of a CNS inflammation impels us to diagnose a definitive IgLON5 autoimmune encephalitis, and eliminates the consideration of IgLON5 antibodies as an epiphenomenon. Two other main differential diagnosis were initially evaluated. First, we initially considered whether this patient might have prodromal Lewy-body dementia with mild cognitive impairment, as he presented with figural memory impairment. However, what argued against the LBD assumption was his lack of core symptoms such as parkinsonism, lack of rapid eye movement (REM)-sleep behavior disorder (RBD) or REM-sleep without atonia (RWA), and his failure to exhibit fluctuating cognition. The lack of RBD and RWA was later confirmed polysomnographically (PSG). Second, cerebrovascular disease was another potential candidate causing cognitive dysfunction, as his MRI had revealed periventricular white matter lesions that might imply cerebrovascular disease, but could also be attributable to the underlying autoimmune process. However, having identified anti-IgLON5 autoantibodies within the CSF finally confirmed the diagnosis of IgLON5-autoimmune encephalitis. Further evidence for an autoimmune-based mechanism in our IgLON5 syndrome is derived from the known association between IgLON5 antibodies and human leukocyte antigen (HLA) class II (14). In addition, the subcortical right frontal cortex lesions, periventricular white matter lesions and mildly generalized atrophy on brain MRI is another potential microstructural indication of an underlying autoimmune process *via* a disturbed blood-brain barrier and the infiltration of IgLON5 autoantibodies. However, no specific IgLON5 lesions have been detected in MRI (**Figures 2A–D**). Furthermore, and although it is much less likely, a concomitant vascular pathology as differential diagnosis cannot be entirely ruled out as an additional cause for these structural brain abnormalities.

From already published data and the present case, it seems likely that IgLON5 antibody-associated autoimmune encephalitis is under-diagnosed and that affected patients are going untreated despite the symptoms’ potential (at least partial) reversibility under therapy, as various studies have shown (2, 9, 11, 15). Although self-reported improved memory is a low-evidence parameter for the efficacy of corticosteroids, lessened axonal neuropathy, no longer detectable ataxia of extremities, and less pronounced sleeping dysfunction at follow-up seem to reveal additional evidence favoring the efficacy of corticosteroids in our case. Patients presenting with cognitive impairment and IgLON5 syndrome often seem to experience better outcomes (15, 16)—an observation in line with our patient’s good response to therapy so far. However, the proof of CSF IgLON5 autoantibodies and signs of CNS inflammation at follow-up support an ongoing disease state. This patient’s follow-up status must therefore be carefully observed, and should his clinical condition turn progressively worse, we would consider a rationale for escalating his therapy, i.e., *via* plasmapheresis. Azathioprine and mycophenolate mofetil are also known to be effective in long-term treatment for IgLON5 encephalitis (15). Nevertheless, noteworthy is our case report’s inherent limitation as a single case report that enables only a low evidence level.

Taken together, even in patients presenting with subtle cognitive impairments in conjunction with sleeping dysfunction, a more specific diagnostic approach including neuropsychological assessment and CSF analysis should be taken to detect IgLON5 positive-encephalitis as a relevant differential diagnoses. The good responsivity that this patient’s cognitive impairment revealed is in line with the literature on IgLON5 syndrome (15) and concurs with our patient’s encouraging prognosis.

## Data Availability Statement

The datasets generated for this study are available on request to the corresponding author.

## Ethics Statement

The study involving a human participant was reviewed by the ethical committee of the University Medical Center Goettingen. The patient provided his written informed consent to participate in this study. Written informed consent was obtained from the individual for the publication of any potentially identifiable images or data included in this article.

## Author Contributions

NH and CB wrote the manuscript. AM, CB, HF, IZ, JW, ME, NH, RV, SH, WS revised the manuscript for important intellectual content. All authors contributed to the article and approved the submitted version.

## Conflict of Interest

The authors declare that the research was conducted in the absence of any commercial or financial relationships that could be construed as a potential conflict of interest.

## References

[1] Gaig C Graus F Compta Y Högl B Bataller L Brüggemann Clinical manifestation of anti-IgLON5 disease. Neurology (2017) 88:1736–43. 10.1212/WNL.0000000000003887 PMC5409845 28381508

[2] Honorat JA Komorowski L Josephs KA Fechner K St Louis EK Hinson SR IgLON5 Antibody: Neurological Accompaniments and Outcomes in 20 Patients. Neurol Neuroimunol Neuroinflamm (2017) 4:e385. 10.1212/NXI.0000000000000385 PMC5515599 28761904

[3] Sabater L Gaig C Gelpi E Bataller L Lewerenz J Torrez-Vega E A novel non-rapid-eye movement and rapid-eye-movement parasomnia with sleep breathing disorder associated with antibodies to IgLON5: a case series, characterisation of the antigen, and post-mortem study. Lancet Neurol (2014) 13:575–86. 10.1016/S1474-4422(14)70051-1 PMC4104022 24703753

[4] Gaig C Iranzo A Santamaria J Graus F The Sleep Disorder in Anti-lgLON5 Disease. Curr Neurol Neurosci Rep (2018) 18:41. 10.1007/s11910-018-0848-0 29796717

[5] Gaig C Iranzo A Cajochen C Vilaseca I Embid C Dalmau J Characterization of the sleep disorder of anti-IgLON5 disease. Sleep (2019) 42(9):zsz133. 10.1093/sleep/zsz133 31198936

[6] Iranzo A Sleep and neurological autoimmune diseases. Neuropsychopharmacology (2020) 45:129–40. 10.1038/s41386-019-0463-z PMC6879573 31302665

[7] Graus F Titulaer MJ Balu R Benseler S Bien CG Cellucci T A clinical approach to diagnosis of autoimmune encephalitis. Lancet Neurol (2016) 15(4):391–404. 10.1016/S1474-4422(15)00401-9 26906964 PMC5066574

[8] Macher S Zimprich F De Simoni D Höftberger R Rommer S Management of autoimmune encephalitis: an observational monocentric study of 38 patients. Front Immunol (2018) 9:2708. 10.3389/fimmu.2018.02708 30524441 PMC6262885

[9] Logmin K Moldovan AS Elben S Schnitzler A Groiss SJ Intravenous immunoglobulins as first-line therapy for IgLON5 encephalopathy. J Neurol (2019) 266:1031–3. 10.1007/s00415-019-09221-3 30737575

[10] Montagna M Amir R De Volder I Lammens M Huyskens J Willekens B IgLON5-associated encephalitis with atypical brain magnetic resonance imaging and cerebrospinal fluid changes. Front Neurol (2018) 9:329. 10.3389/fneur.2018.00329 29867738 PMC5966542

[11] Simabukuro MM Sabater L Adoni T Cury RG Haddad MS Moreira CH Sleep disorder, chorea, and dementia associated with IgLON5 antibodies. Neurol Neuroimmunol Neuroinflamm (2015) 2:e136. 10.1212/NXI.0000000000000136 26236762 PMC4516399

[12] Witt JA Coras R Schramm J Becker AJ Elger CE Blümcke I The overall pathological status of the left hippocampus determines preoperative verbal memory performance in left mesial temporal lobe epilepsy. Hippocampus (2014) 24:446–54. 10.1002/hipo.22238 24375772

[13] Erro ME Sabater L Martínez L Herrera M Ostolaza A de Gurtubay IG Anti-IGLON5 disease. Neurol Neuroimmunol Neuroinflamm (2020) 7:e651. 10.1212/NXI.0000000000000651 31826985 PMC7007636

[14] Gaig C Ercilla G Daura X Ezquerra M Fernández-Santiago R Palou E HLA and microtubule-associated protein tau H1 haplotype associations in anti-IgLON5 disease. Neurol Neuroimmunol Neuroinflamm (2019) 6(6):e605. 10.1212/NXI.0000000000000605 31454761 PMC6705627

[15] Cabezudo-García P Mena-Vázquez N Estivill Torrús G Serrano-Castro P Response to immunotherapy in anti-IgLON5 disease: a systematic review. Acta Neurol Scan (2019) 141(4):263–70. 10.1111/ane13207 31853949

[16] Brunetti V Della Marca G Spagni G Iorio R Immunotherapy improves sleep and cognitive impairment in anti-IgLON5 encephalopathy. Neurol Neuroimmunol Neuroinflamm (2019) 6(4):e577. 10.1212/NXI.0000000000000577 31355313 PMC6624089

