# Corrigendum: Figural Memory Impairment in Conjunction With Neuropsychiatric Symptoms in IgLON5 Antibody-Associated Autoimmune Encephalitis

**DOI:** 10.3389/fpsyt.2020.589466

**Published:** 2020-09-11

**Authors:** Niels Hansen, Sina Hirschel, Winfried Stöcker, Anja Manig, Hannah Sönne Falk, Marielle Ernst, Ruth Vukovich, Inga Zerr, Jens Wiltfang, Claudia Bartels

**Affiliations:** ^1^ Department of Psychiatry and Psychotherapy, University Medical Center Goettingen, Goettingen, Germany; ^2^ Euroimmun Laboratory, Seekamp, Luebeck, Germany; ^3^ Department of Clinical Neurophysiology, University Medical Center Goettingen, Goettingen, Germany; ^4^ Department of Neuroradiology, University Medical Center Goettingen, Goettingen, Germany; ^5^ Neurochemical Laboratory, Department of Neurology, University Medical Center Goettingen, Goettingen, Germany; ^6^ German Center for Neurodegenerative Diseases, Goettingen, Germany; ^7^ Neurosciences and Signalling Group, Department of Medical Sciences, Institute of Biomedicine, University of Aveiro, Aveiro, Portugal

**Keywords:** encephalitis, IgLON5 antibodies, memory impairment, figural memory, autoimmunity

In the original article, we overstayed to include the funder **“**Open Access Publication Fund of the Georg-August-University of Göttingen”. A correction has been made to the **Funding**:

“This work was supported by the Open Access Publication Fund of the Georg-August-University of Göttingen.”

In the original article, there was a mistake in **Table 1** as published. The reference values for Aß1-42 and Aß ratio were incorrectly reported in **Table 1**. Furthermore, the displayed unit of Aß ratio was incorrect in **Table 1**. **The correct reference values in** **Table 1** **are:** **Aß1-42 pg/ml (pathological: < 450 pg/ml) and Aß ratio (pathological: < 0.5).** The correct reference values of Aß1-42 and Aß ratio and the corrected **Table 1** appear below.

**Table 1:** Results of individual investigations at first visit and follow-up.

Parameter	First visit	Follow-up
**CSF analysis**		
Cell count /ul (pathological: >5 µl)	8	8
Lymphocytes in %	93	92
Monocytes in %	5	.
Plasma cells in %	2	2
Albumin mg/L	518	615
IgG mg/L	69.3	62.5
IgA mg/L	10.7	9.6
IgM mg/L	0.97	0.84
QAlb %	11.5	15.4
QIgG %	7.3	8.9
QIgA %	4.3	5.2
QIgM %	2.4	2.4
Lactat mmol/L	2	2
Oligoclonal IgG	–	–
NSE ng/ml (pathological: >30 ng/ml)	19.3	.
S100 µg/ml (pathological: >2.7 µg/ml)	2	.
Tau pg/ml (pathological: >450 pg/ml)	409	.
P-Tau 181 pg/ml (pathological: > 61 pg/ml)	68	.
Aß1-42 pg/ml (pathological: < 450 pg/ml)	715	.
Aß1-40 pg/ml	9565	.
Aß ratio (pathological: < 0.5)	0.75	.
**Serum**		
Oligoclonal IgG	–	–
CRP mg/l	42.5	1.1
Leukocytes 10^3^ µl	5.63	4.29
Neuropsychological testing*		
MMSE	30	29
CDT	01	01
CERAD Boston Naming Test	PR 31	PR 82
CERAD semantic fluency	PR 50	PR 66
CERAD phonemic fluency	PR 82	PR 62
TMT part A	PR 76	PR 99
WAIS-IV Digit Symbol Test	PR 84	PR 95
TMT division part B/A	PR 84	PR 58
RWT semantic fluency (alternating)	PR 30	PR 75
RWT phonemic fluency (alternating)	PR 63	PR 54
WAIS-IV Digit Span forward	PR 05*	PR 05*
WAIS-IV Digit Span backward	PR 16	PR 16
CERAD List Learning (trials 1-3)	PR 34	PR 34
CERAD List Recall (savings)	PR 46	PR 54
CERAD List Recognition/discriminibility	PR 21	PR 79
WMS-IV Logical Memory I	PR 50	PR 50
WMS-IV Logical Memory II	PR 16	PR 50
CERAD Figure recall (savings)	PR 01*	PR 12*
WMS-IV Visual Reproduction I	PR 25	PR 50
WMS-IV Visual Reproduction II	PR 50	PR 75
CERAD Figure Copy	PR 73	PR 18
ROCFT Copy	PR>16	PR>16
WAIS-IV Block Design	PR 25	PR 75
**Psychopathology**		
BDI-II	04	05
**Polysomnography**		
Apnoe-Hypopnoe-Index	.	9.7
PLMI	.	64.6
Basal 0_2_ desaturation %	.	94
Minimal 0_2_ desaturation %	.	89.0
0_2_ desaturation index %	.	13

*For better comparison, cognitive data is presented as percentiles (PR) unless otherwise indicated. Asterisk denote performance below the normal range (PR<16). . = data not available. - = not present. Polysomnography data are derived from the second night. AHI, apnoe-hyperpnoe index; Aß ratio, ß amyloid 1-42/1-40 ratio; Aß1-40, ß amyloid 1-40; Aß1-42, ß amyloid 1-42; BDI-II, Beck Depression Inventory; CSF, cerebrospinal fluid; IgA, immunoglobulin A; IgG, immunoglobulin G; IgM, immunoglobulin M; MMSE, Mini-Mental Status Examination; CDT, Clock Drawing Test; CERAD, Consortium to Establish a Registry for Alzheimer’s Disease; TMT, Trail Making Test; RWT, ROCFT, Rey-Osterrieth-Complex-Figure test; WAIS-IV, Wechsler Adult Intelligence Scale IV; WMS-IV, Wechsler Memory-Scale IV, NSE, neuron specific enolase; O2 desaturation, oxygen desaturation; PLMI, periodic limb movement index; P-tau181, phosphorylated tau protein 181; QAlb, quotient albumin; QIgA, quotient immunoglobulin A; QIgG, quotient immunoglobulin G; QIgM, quotient immunoglobulin M; T-tau, total tau protein.

The authors make an apology for these errors. In addition, the authors declare that this does not alter the scientific conclusions of the article.

